# Introduction: Open Dialogue around the world – implementation, outcomes, experiences and perspectives

**DOI:** 10.3389/fpsyg.2022.1093351

**Published:** 2023-01-10

**Authors:** David Mosse, Raffaella Pocobello, Rob Saunders, Jaakko Seikkula, Sebastian von Peter

**Affiliations:** ^1^Department of Anthropology and Sociology, SOAS University of London, London, United Kingdom; ^2^Institute of Cognitive Sciences and Technologies, National Research Council, Rome, Italy; ^3^CORE Data Lab, Centre for Outcomes Research and Effectiveness (CORE), University College London, London, United Kingdom; ^4^Department of Psychology, University of Jyväskylä, Jyväskylä, Central Finland, Finland; ^5^Department of Psychosocial Health, University of Agder, Kristiansand, Vest-Agder, Norway; ^6^Medical School Brandenburg, Neuruppin, Germany

**Keywords:** Open Dialogue, mental health, implementation, practices, outcomes, healthcare systems, research methods, global

## Introduction

There is an urgent need for innovative and alternative approaches in global mental healthcare systems given problems such as low rates of functional recovery, long-term dependence on psychoactive medication, pressure on hospital beds and crisis services, long wait-times, staff burnout and dissatisfaction from service users and their families. This Frontiers Research Topic focuses on Open Dialogue, a mental healthcare approach which has the potential to address some of these challenges and that is gaining worldwide momentum.

As this Research Topic will explore, Open Dialogue takes different forms across varied healthcare contexts but nonetheless has a core philosophy, values and set of principles. These were first developed and applied in Finland (Western Lapland) through a complementary process of therapeutic innovation and research over 40 years (Alakare and Seikkula, 2022). Open Dialogue brought change in local healthcare at two levels. First, a culture of dialogical communication between staff, service users and caregivers was established. Instead of an expert-led diagnosis-treatment model, service users and members of their social network were placed at the center of a dialogical process aimed at discovering ways out of crisis. Second, community-based, multi-disciplinary teams were organized to provide immediate help in crisis, accommodating service user and family needs, continuity of support by the same team and a primarily psychotherapeutically-oriented approach (minimizing medication use). These were the key emerging principles of Open Dialogue that have been further elaborated upon during the past decades (Olson et al., 2014).

Open Dialogue emphasizes the practitioners' capacity for empathy, presence and listening. It avoids interpreting others' experience through symptom-focused diagnoses. Instead, Open Dialogue encourages listening to what individuals and their families (or other kinds of social network) say about difficult experiences and events that have happened to them. It attends to words and meanings spoken in the different voices of those who participate in Open Dialogue “network meetings” (Seikkula et al., 1995). These meetings are where important care decisions are made, openly and with those who are the focus of concern. Open Dialogue is thus based on a mental healthcare epistemology that prioritizes everyday relationships and context-bound understandings over clinical diagnosis; “being with” rather than “doing to”. Transparency is important: all information is shared, and all voices are heard, thereby recognizing diversity and attempting to mitigate the effect of power differentials during the process of support.

This approach has implications for the organization of services to ensure immediate response to crisis, flexible and continuous care, and to enable work with multiple people in treatment systems which otherwise have an individualistic paradigm (Tse and Ng, 2014). Increasingly, Open Dialogue teams include people with lived experience as “peer” practitioners; but there is also a general expectation that the approach requires the skilled use of personal experience and emotions in dialogical encounters (Grey, 2019). This challenges conventional ideas on the source of clinical knowledge and definitions of expertise, changing established professional self-understandings and claims (von Peter et al., 2021). There are implications for clinical governance including responses to risk, clinical note-taking, discharge, flexible working and the boundaries around clinical work, as well as for training and supervision (Buus et al., 2021).

While presenting challenges to the conventional organization of mental healthcare, Open Dialogue has attracted attention from leaders and policymakers in different countries because of growing evidence from studies (initially from Western Lapland) for the effectiveness of the approach. Early research in Finland (Seikkula, 1991) “confirmed that immediate help, with the flexible involvement of the service user and their network, along with psychological continuity, were key factors in reducing the need for hospitalization” (Alakare and Seikkula, 2022, p. 47). The approach was subsequently found to be associated with reduced use of neuroleptic medication, maintenance of recovery from acute psychosis and return to education or employment (Seikkula et al., 2006, 2011; Aaltonen et al., 2011; Alakare and Seikkula, 2022). Research suggests not only that the experience of receiving (and delivering) care is improved, but also healthcare costs are reduced by Open Dialogue through faster recovery, reduced hospitalization, less time in treatment and reduced need for disability benefits (Bergström et al., 2018).

Alongside effectiveness, the ethical dimensions of Open Dialogue – justice, dignity, compassion – have promoted commitment to the approach. Open Dialogue is aligned to mental healthcare which is trauma-informed, and its person-centered and rights-based (von Peter et al., 2019) approach has recently been recognized as a “good practice” example of crisis services, promoting rights and recovery in the World Health Organization's “Guidance on community mental health services” (WHO, 2021). Open Dialogue also features in the Council of Europe's compendium of good practices intended to eliminate coercive practices in mental health settings as a matter of human rights (Council of Europe, 2021).

## Why this research topic?

Open Dialogue is now practiced in various regions globally, in more than 24 countries, including several in Europe as well as Australia, Japan, India, Latin America and the United States (Pocobello, 2021). While originally a public sector service, Open Dialogue has now found applications in NGOs, associations and private practice. Services also vary regarding the groups they engage and the social context. Open Dialogue services have different inclusion and exclusion criteria. For instance, some exclude and others include people with learning difficulties (Fredman and Lynggaard, 2015); similarly in relation to people with drug or alcohol problems.

Relatively little is known about the practice and effectiveness of Open Dialogue in these different settings, and whether findings from the original studies in Finland are replicated. The question of how differences in the form and delivery of Open Dialogue might impact outcomes is crucial as Open Dialogue is adapted to local healthcare systems and contingencies. In view of the emerging diversity, it is an empirical question whether Open Dialogue is a clearly demarcated intervention or a broad approach manifest in a variety of local forms.

This Research Topic on “Open Dialogue Around the World – Implementation, Outcomes, Experiences and Perspectives” opens a window on the range and scope of research exploring different aspects and contexts of Open Dialogue. Through its inclusive set of contributions, the Research Topic aims to serve as a bridge between research and clinical practice. Indeed, the Open Dialogue approach is a system of care that has developed through its constant connection with ongoing research on the practice.

The Research Topic contributes to various kinds of inquiry that are currently the focus of Open Dialogue research and practice:

- At the broadest level, the results of an international survey of Open Dialogue services are presented, and the diverse variants of this approach and its organization within health care systems.
- Country or healthcare system-specific organizational studies provide case-studies, and comparisons of Open Dialogue services invited from across the globe. These present not only the adaptations to the initial incarnation of Open Dialogue, but also discuss the challenges to sustaining Open Dialogue practice in different healthcare systems. These organizational studies highlight the healthcare bureaucracies to which Open Dialogue has to adapt, including systems of clinical governance, risk management, performance indicators and professional hierarchies. Open Dialogue can also bring institutional change through sometimes radically different notions of accountability. Here, Open Dialogue is understood in its political dimension: a reflection on institutional power and a movement for change, responding to the experience and demands of individuals, families and communities who may have had testing experiences of psychiatric systems.
- Further understanding is provided from studies on the internal organization and functioning of Open Dialogue services, including their particularity and distinctiveness. Accounts of training and supervision in Open Dialogue are valuable, both to describe service organization and also portray the subjective experience of trainers and trained. Accounts of Open Dialogue training continue to highlight its principles and their cultivation in terms of dialogical capacities such as listening, presence, embodiment, forms of questioning and reflection and the varied practices of presence, such as mindfulness that are incorporated into training. Investigation into the experience and organizational conditions of peer work in Open Dialogue – the opportunities and contradictions in different service structures – is also a growing area of inquiry (Razzaque and Stockmann, 2016; Grey, 2019) to which this Research Topic contributes.
- This Research Topic contains studies on Open Dialogue as a therapeutic process. Here research is accumulating fine-grained accounts of dialogical interactions and the meaning-making out of crisis. Since Open Dialogue is a social network approach, the relational dynamics of Open Dialogue's “network meetings” and their impact are of interest. The encouragement of different voices and viewpoints of participants (the “polyphony”) and the way the truths of persons at the center and their family members come into dialogue with psychiatric knowledge, diagnosis and decision-making are productive fields of inquiry. The nature of Open Dialogue networks and family (or multi-family) involvement and the relational dynamics that unfold need to be understood. They are shaped by family systems and social-cultural environments in ways that are being discovered through Open Dialogue practice, and include particular challenges such as where relationships involve violence. As Open Dialogue diversifies into different settings, the affordances of cultural identity, kinship systems, different embodied, symbolic and linguistic repertoires come into play in collaborative meaning-making and fostering social connection that is involved in moving from crisis to recovery.
- Evidence on the outcomes of Open Dialogue is important for the status of Open Dialogue in global healthcare systems. The world's first large-scale multi-site cluster randomized controlled trial of Open Dialogue in the UK (ODDESSI) is under way and investigates the effectiveness of Open Dialogue within the UK's National Health Service (NHS) in comparison with established treatment models (Pilling et al., 2022). In parallel, randomized-controlled studies of Open Dialogue are being undertaken in other countries/health systems, across different statutory services and health insurance companies. Other non-randomized studies have focused on specific outcomes such as psychotropic medication prescribing across Open Dialogue/non-Open Dialogue client groups (in Finland) (Alakare and Seikkula, 2022). An international feasibility study named HOPEnDialogue is currently underway, and it aims to establish an evaluation framework to assess the outcomes of Open Dialogue internationally (Alvarez et al., 2021).
- This Research Topic pays attention to the fact that Open Dialogue services have been investigated through a range of research methodologies (Freeman et al., 2019; Buus et al., 2021), including multi-site observational studies used to test feasibility and efficacy (Harding et al., 1987; Seikkula et al., 2006). Open Dialogue experiences and outcomes have been studied through various survey instruments, including service-user (and family/social network member) self-report scales (e.g., quality of life, or perceived satisfaction with network sessions/service in general). Open Dialogue “key elements” criteria have been developed against which clinician adherence and organizational fidelity can be measured (Olson et al., 2014). Methods to evaluate Open Dialogue other than structured questionnaires measuring outcomes or adherence include descriptive case-studies of services or organizations and client case reports (or samples of these).

Assessing the process of Open Dialogue rather than outcomes *per se*, has brought in a range of qualitative methods such as focus group discussions (with clients and clinicians), recorded practitioner dialogues, team/peer group reflections, practitioner evaluative self-descriptions, subjective reflections and personal experience narratives (Rober, 2005; Gromer, 2012; Bøe et al., 2015; Cubellis, 2020; Dawson et al., 2021). Some Open Dialogue studies are framed as action-research to capture unfolding Open Dialogue programmes (Hopper et al., 2020), and long-term team-based ethnographic research by anthropologist-practitioners offers deep qualitative insight into Open Dialogue processes and effects (Pope et al., 2016; Cubellis, 2022; Mosse, in press). This research involves a phenomenological orientation to Open Dialogue, including attention to its emotional and embodied aspects, as well as the social, institutional and material context of Open Dialogue services (Cubellis et al., 2021).

- Alongside empirical studies, conceptual work has always been central to research and contributes to a still-nascent theory of Open Dialogue (Andersen, 1996; Seikkula, 2003; Seikkula et al., 2003; Shotter, 2011). Such analytical and philosophical reflections are not limited to viewing Open Dialogue in its own terms, but equally in relation to antecedent or adjacent therapeutic orientations whether systemic family therapy or psychoanalysis, both of which have influenced Open Dialogue.

## Conclusion

Current Open Dialogue research and clinical practice, the breadth and depth of which is demonstrated in this Frontiers Research Topic, not only provide some answers to established to questions but also frame new ones. Much is yet to be discovered about Open Dialogue and the individual and institutional transformations it may entail. Questions on the salient core elements, the relevant variables, the institutional preconditions or barriers, the contextual factors of a given locality, client population or clinician group need to be constantly re-visited, while Open Dialogue as a field of therapeutic intervention spreads and diversifies across the globe. Gaining and sharing relevant knowledge requires active incorporation of research from different perspectives and subject positions including that of researchers and practitioners, clinicians and clients, peers, survivors and user researchers, and varied forms of collaborations alongside the multiplication of Open Dialogue across countries, sites and services.

## Data availability statement

The original contributions presented in the study are included in the article/supplementary material, further inquiries can be directed to the corresponding author.

## Author contributions

DM wrote the first outline draft of the article, based on contributions from other authors. All authors contributed to the development, finalization, and approval of the article.
